# Fallopian Tube Herniation: An Unusual Complication of Surgical Drain

**DOI:** 10.1155/2012/194350

**Published:** 2012-08-02

**Authors:** Lipi Sharma, Alpana Singh, Sruthi Bhaskaran, A. G. Radhika, Gita Radhakrishnan

**Affiliations:** UCMS and GTBH, Dilshad Garden, New Delhi 110095, India

## Abstract

*Background*. Surgical drains have been used since time immemorial, but their use is not without complications. By presenting this case we aim to describe an uncommon complication of herniation of fallopian tube following the simple procedure of surgical drain removal. *Case Presentation*. This case describes a 23-year G2P1L1 who underwent an emergency cesarean section for obstructed labor with intraperitoneal drain insertion. The patient had an uneventful postoperative period, drain was removed on day 4, and she was discharged. She presented on day 8 with the complaint of soakage of drain site dressing. On examination an edematous, tubular structure with early sign of necrosis was seen coming out of drain site and a provisional diagnosis of appendix herniation was made. On emergency laparotomy fallopian tube was seen coming out through the drain site and salphingectomy was done. *Conclusion*. Drains are not a substitute for good surgical technique. Although herniation of intestine, omentum, appendix, gall bladder, and ovary have been reported, we could not find any case of fallopian tube herniation in the literature searched by us.

## 1. Background

Surgeons use drain to prevent intra-abdominal fluid collection, both therapeutically and prophylactically. Surgical drains, as useful as they are, have been noted not to be without complications; such as, hemorrhage, infection, tissue damage, pain, blockage, and herniation of viscera [1]. In the case illustrated below, fallopian tube (a rare possibility) herniated out of surgical drain site, which due to delay in detection lead to salphingectomy.

## 2. Case Report

A 23-year-old, unbooked, G2P1L1 presented to the obstetric emergency at term with history of pain in abdomen since morning and leaking per vaginum since 6 hrs. On examination she was of average built. Her pulse was 106/min and blood Pressure was 100/70 mm Hg. On per abdominal examination her uterus corresponded to 34 weeks size, the lower uterine segment was stretched out, and fetus was in transverse lie. The uterus was having mild contractions and fetal heart sound was absent. On per vaginum examination vagina was hot and dry, cervix was 5 cms dilated, fully effaced, and membranes were absent with fetal hand felt in vagina. An emergency caesarean section was done in view of transverse lie with hand prolapse in obstructed labor with intrauterine fetal demise.

Intraoperatively it was found that due to obstruction uterine musculature had become edematous. Due to continuous ooze from lower uterine segment, a drain was inserted from a separate incision in right iliac fossa and placed intraperitoneally in pouch of Douglas. The patient developed mild cough on day 2; otherwise her postoperative period was uneventful. The drain output had reduced to 25 mL by day 4, drain was removed and she was discharged with advice to come on day 8 for stitch removal. At the time of stitch removal patient complained that her drain site dressing was soaked. On examination a 4 × 5 cm pinkish brown, tubular, edematous mass with slough at base was seen coming out through drain site which had become strangulated with early changes of necrosis. It was nontender and nonreducible. The patient gave history of normal bowel movement. On examination her vitals were stable, bowel sounds were present, and there were no signs of peritonitis. Following surgical opinion patient was taken for emergency laparotomy on suspicion of intestinal or appendicular herniation from drain site.

**Figure 1:**
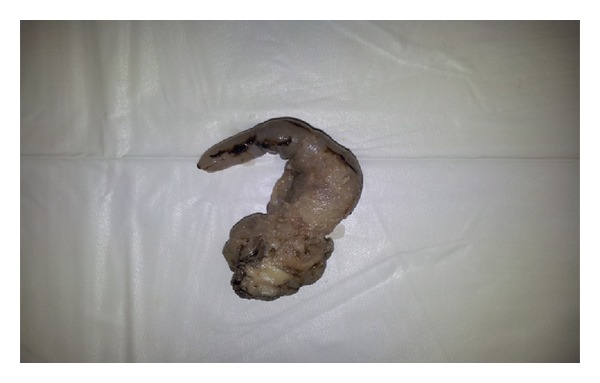
Right Fallopian tube: swollen, edematous with early signs of necrosis.

 Intraoperatively the herniating viscus was the right fallopian tube which has become necrosed due to strangulation, appendix and gut were normal. Right salphingectomy was done and tissue was sent for histopathology. The histopathology reported it as fallopian tube with submucosal edema, congestion, and features of acute inflammation (Figure 1). Postoperative period was uneventful and patient was discharged.

## 3. Discussion

Drains have been used in surgical practice since time immemorial. Indications for surgical drain use may be therapeutic (to evacuate existing collection of fluid) or prophylactic (to prevent the collection of fluid). This fluid may be blood, bile, pus, urine, serum/lymph, pancreatic secretion, and bowl anastomotic leaks. Various reports of fallopian tube as a content of hernia sac (inguinal and femoral hernia) [2], and herniation of fallopian tube posthysterectomy (abdominal and vaginal) [3] are present. There are reports of herniation of intestine [4], appendix [5], omentum [6], gall bladder [7] (single report), and ovary [8] (single report) from surgical drain site but, on reviewing indexed English literature we could not find any report of fallopian tube herniation. What lead to fallopian tube herniation from surgical drain site in this patient and was it preventable?

The incidence of herniation of viscera from port site in laparoscopic surgery is reported to be .65%–2.5% [9]. Do the same factors govern herniation from surgical drain site following open surgery? In our patient a passive, closed Penrose drain (no. 32) was placed prophylactically in pouch of Douglas. Large meta-analyses [10] have revealed that the indications of prophylactic drains should be minimized in case of uncomplicated surgeries. The drain used had side holes which do not have any influence on drainage but lead to tissue entanglement. 

**Figure 2:**
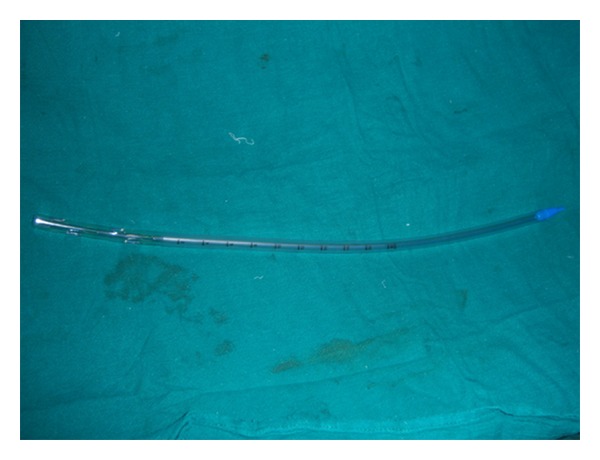
Drainage tube 32 FG.

The drain used (Figure 2) had an external diameter of 10 mm. It has been reported that herniation of viscera increases with increase in port size ≥10 mm [11]. Where ever possible fascial defects of ≥10 mm should be closed. The patient developed cough on postoperative day 3. Recurrent increase in intra-abdominal pressure caused by coughing or straining, prolonged surgery, poor nutrition, wound infection, obesity, and steroid use are known to cause poor healing and herniation [12]. Also, the large size of puerperal uterus had placed the fallopian tube close to the drain site which may be one of the factors which lead to its herniation.

Wrong technique of insertion and removal can be a causative factor but it was not so in this case. We used asymmetrical method which causes peritoneal stretching for insertion of drain rather than using direct stab incision. While removing, gradual sustained pressure should be used to withdraw the drain.

## 4. Conclusion

Surgical drains, although used commonly in elective as well as emergency surgeries, are not without complications. Such complications can be avoided by more restricted use of surgical drains.
